# Supplementary material for: In vivo glucoregulation and tissue-specific glucose uptake in female Akt substrate 160 kDa knockout rats
Source: PLoS One. 2020 Feb 13;15(2):e0223340. doi: 10.1371/journal.pone.0223340 (PMC7018090; doi:10.1371/journal.pone.0223340)

S3 Mitochondrial proteins in hearts collected immediately after the hyperinsulinemic-euglycemic clamp performed in WT (open bars) and KO (filled bars) rats.

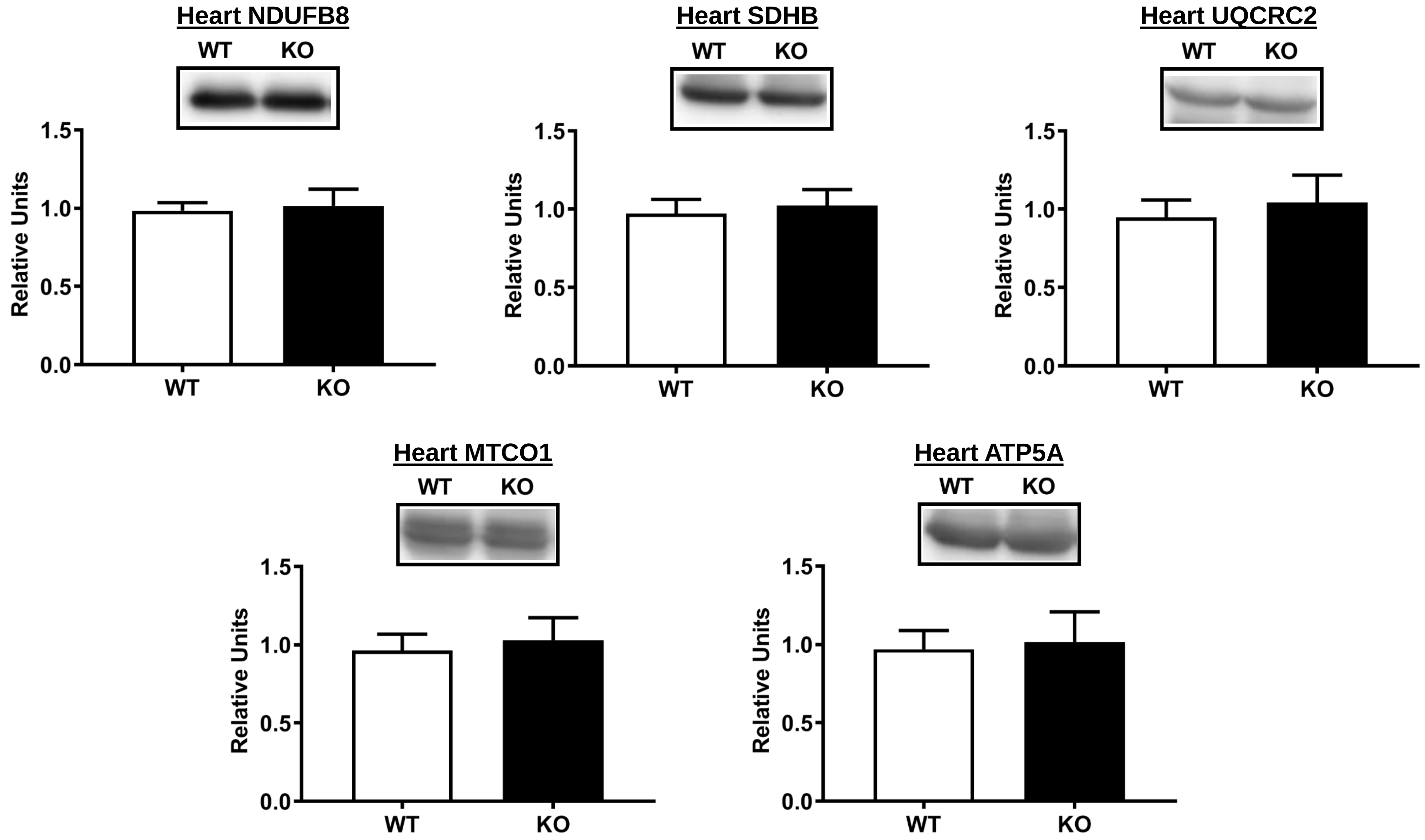

Supplement: S3 Fig — Extensor digitorum longus = EDL. White adipose tissue = WAT. Means ± SEM for 4 rats of each genotype. (PDF) [file pone.0223340.s003.pdf]
