# Supplementary material for: In vivo glucoregulation and tissue-specific glucose uptake in female Akt substrate 160 kDa knockout rats
Source: PLoS One. 2020 Feb 13;15(2):e0223340. doi: 10.1371/journal.pone.0223340 (PMC7018090; doi:10.1371/journal.pone.0223340)
Supplement: S1 Raw images — (PDF) [file pone.0223340.s004.pdf]

Figure 1

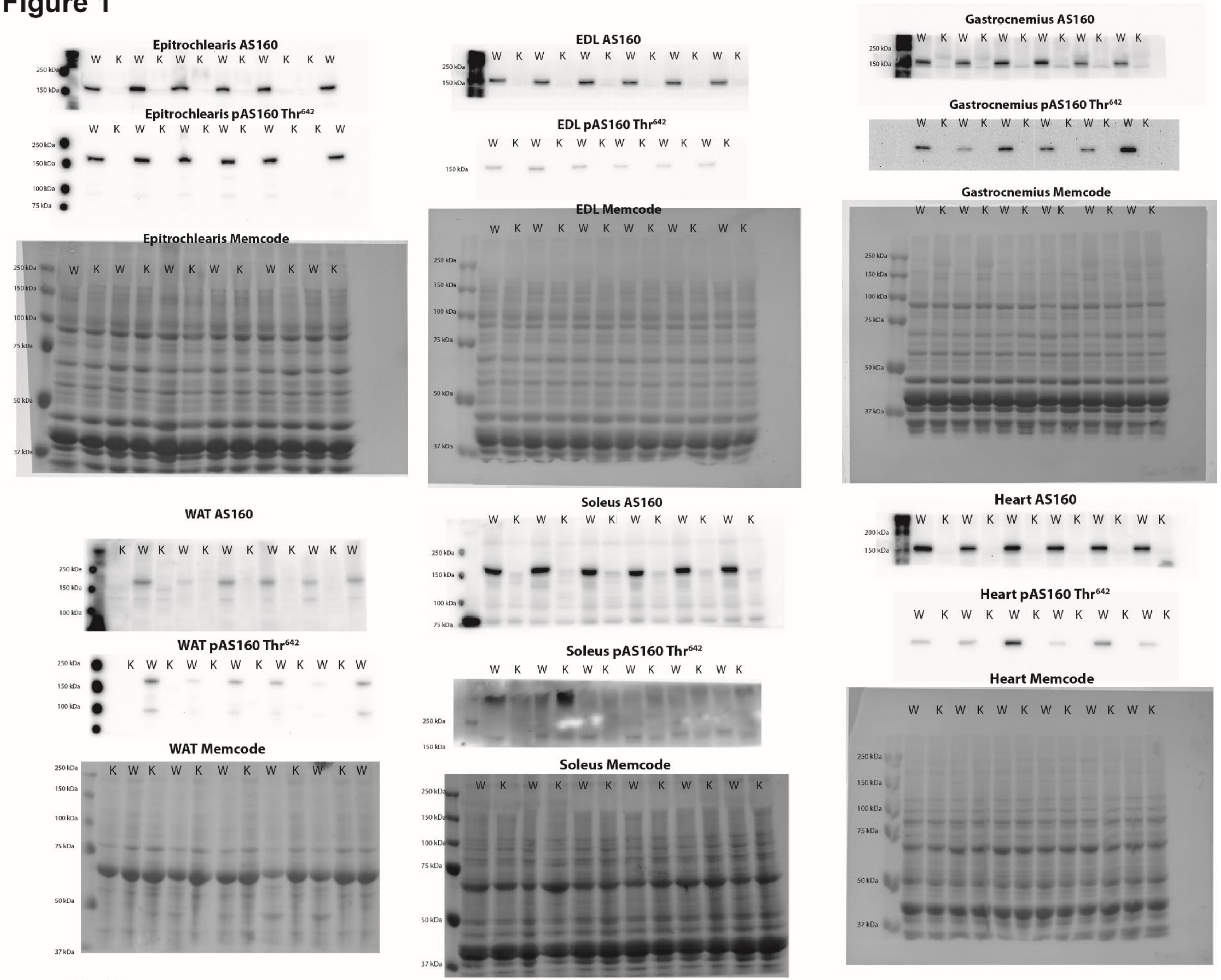

W: wild type
K: AS160 Knock-out
All images were captured using the Chemilluminescence channel by FluorChemE by Proteinsimple

Figure 4 (All samples are from the hyperinsulinemic euglycemic clamp)

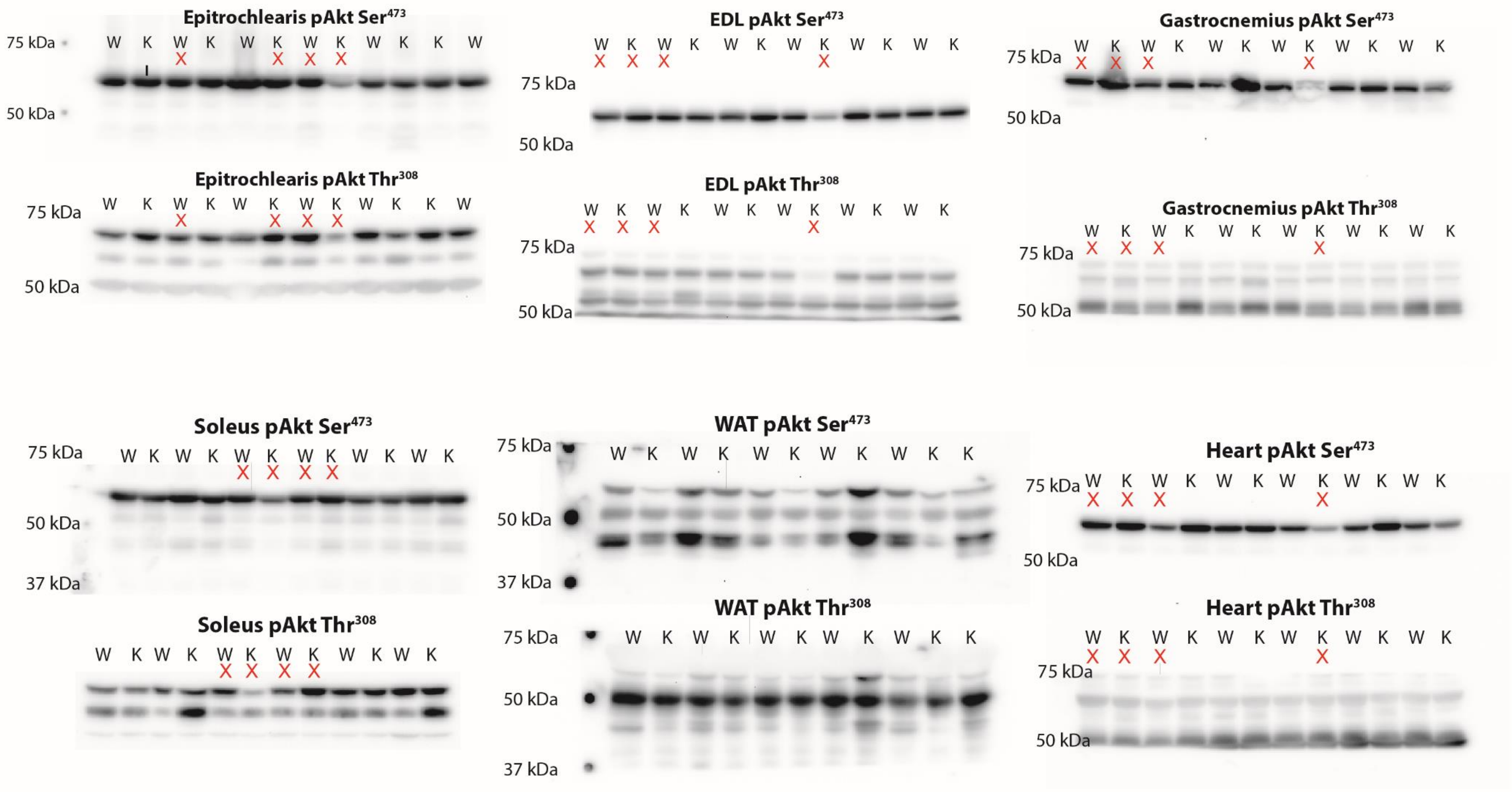

W: wild type
K: AS160 Knock-out
X: value excluded from statistical analysis because of technical problems with the hyperinsulinemic euglycemic clamp
All images were captured using the Chemilluminescence channel by FluorChemE by Proteinsimple

Figure 5 (All samples are from the hyperinsulinemic euglycemic clamp)

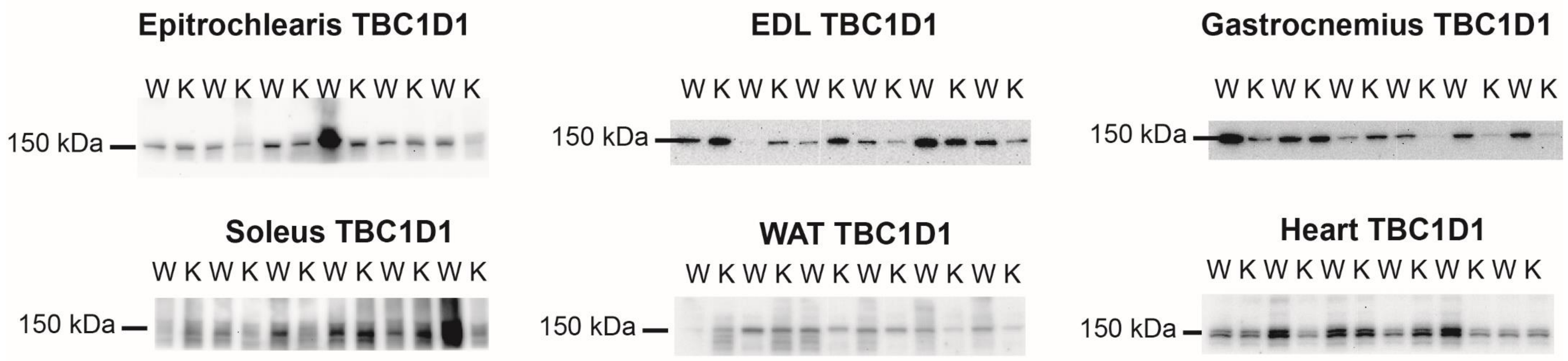

W: wild type
K: AS160 Knock-out
All images were captured using the Chemilluminescence channel by FluorChemE by Proteinsimple

Figure 6 (All samples are from the hyperinsulinemic euglycemic clamp)

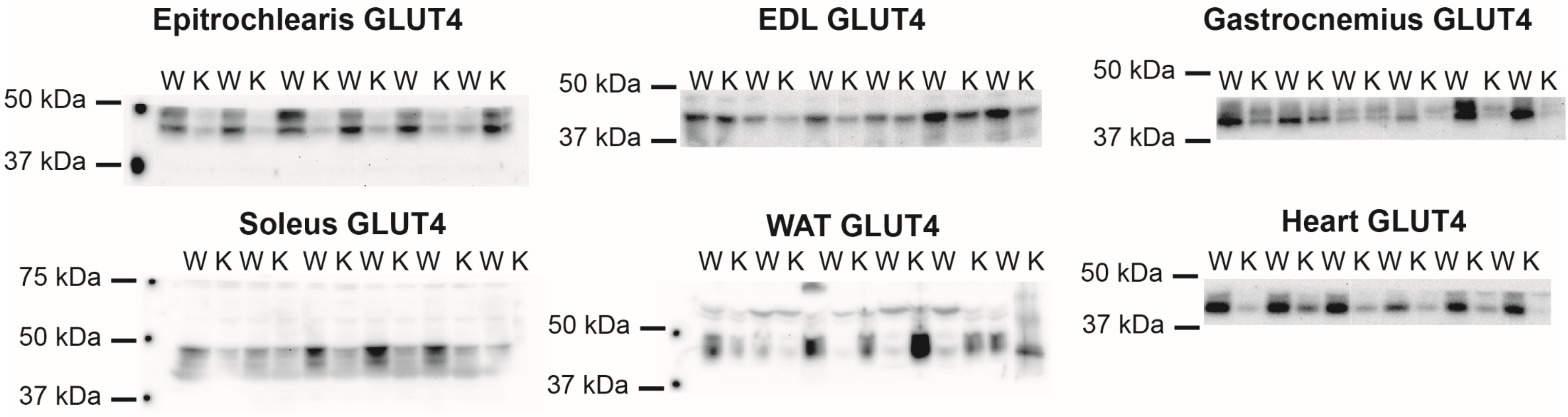

W: WT
K: AS160 Knock-out

All images were captured using the Chemilluminescence channel by FluorChemE by Proteinsimple

Figure 7 (All samples are from the hyperinsulinemic euglycemic clamp)

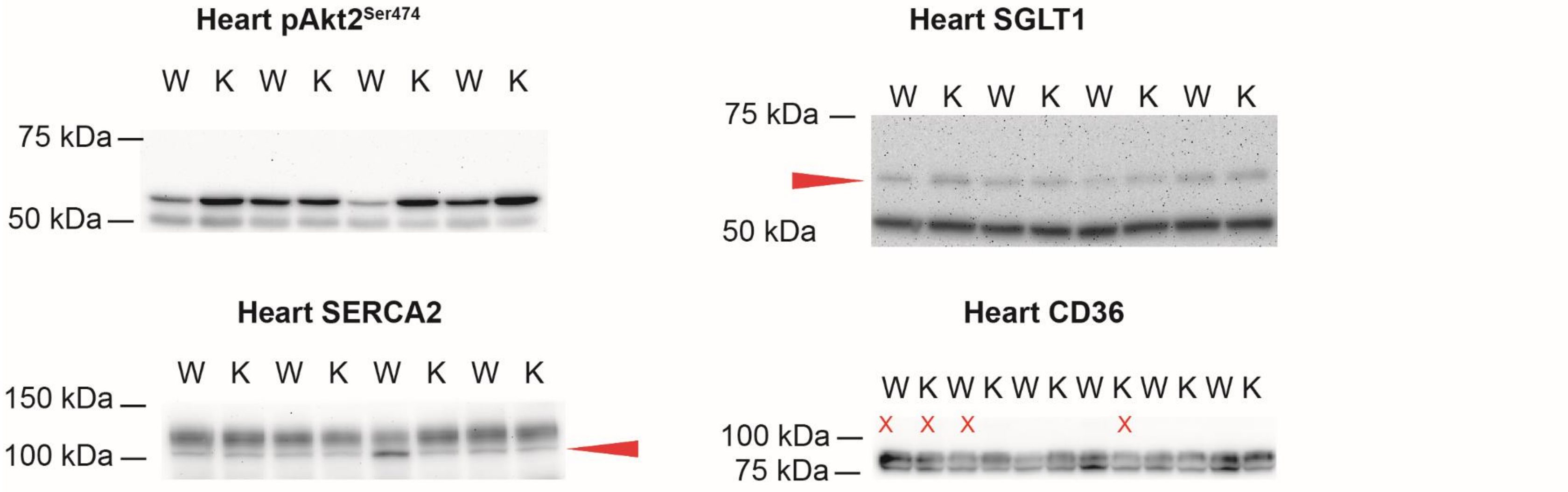

W: wild type
K: AS160 Knock-out
All images were captured under the Chemilluminescence channel by FluorChemE by Proteinsimple
X: value excluded from statistical analysis because of technical problems with the hyperinsulinemic euglycemic clamp
Denotes quantified band at expected molecular weight

Figure 8 (All samples are from the hyperinsulinemic euglycemic clamp)

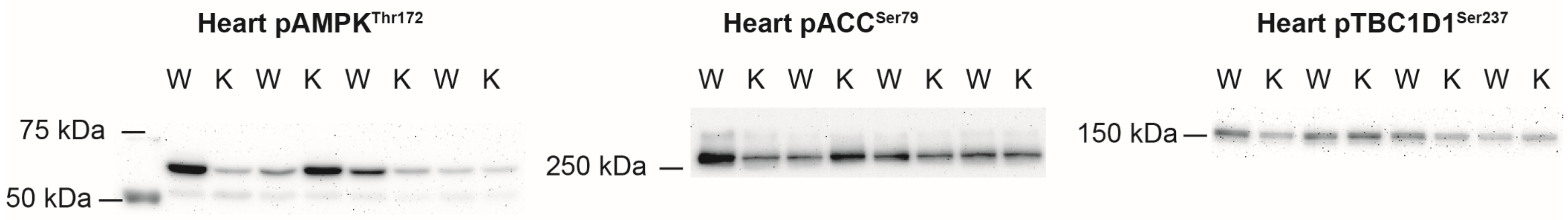

W: wild type
K: AS160 Knock-out
All images were captured using the Chemilluminescence channel by FluorChemE by Proteinsimple

Figure 9 (All samples are from the hyperinsulinemic euglycemic clamp)

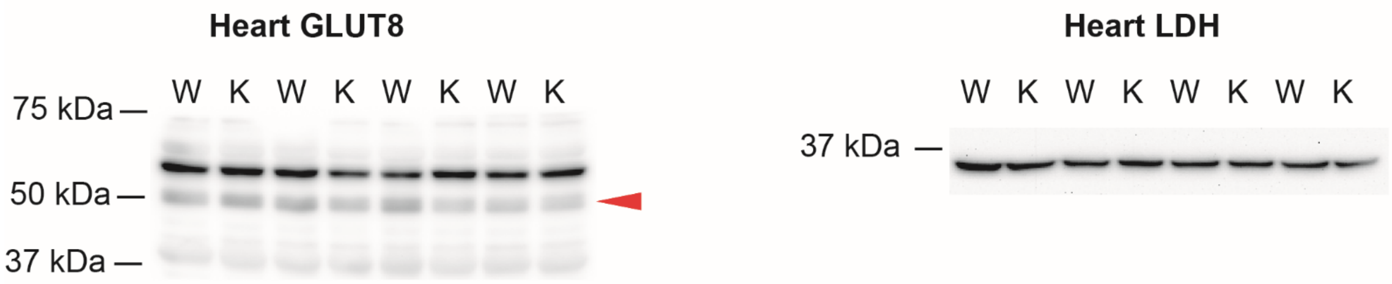

W: wild type
K: AS160 Knock-out
All images were captured using the Chemilluminescence channel by FluorChemE by Proteinsimple
◀ Denotes quantified band at expected molecular weight

Figure 11

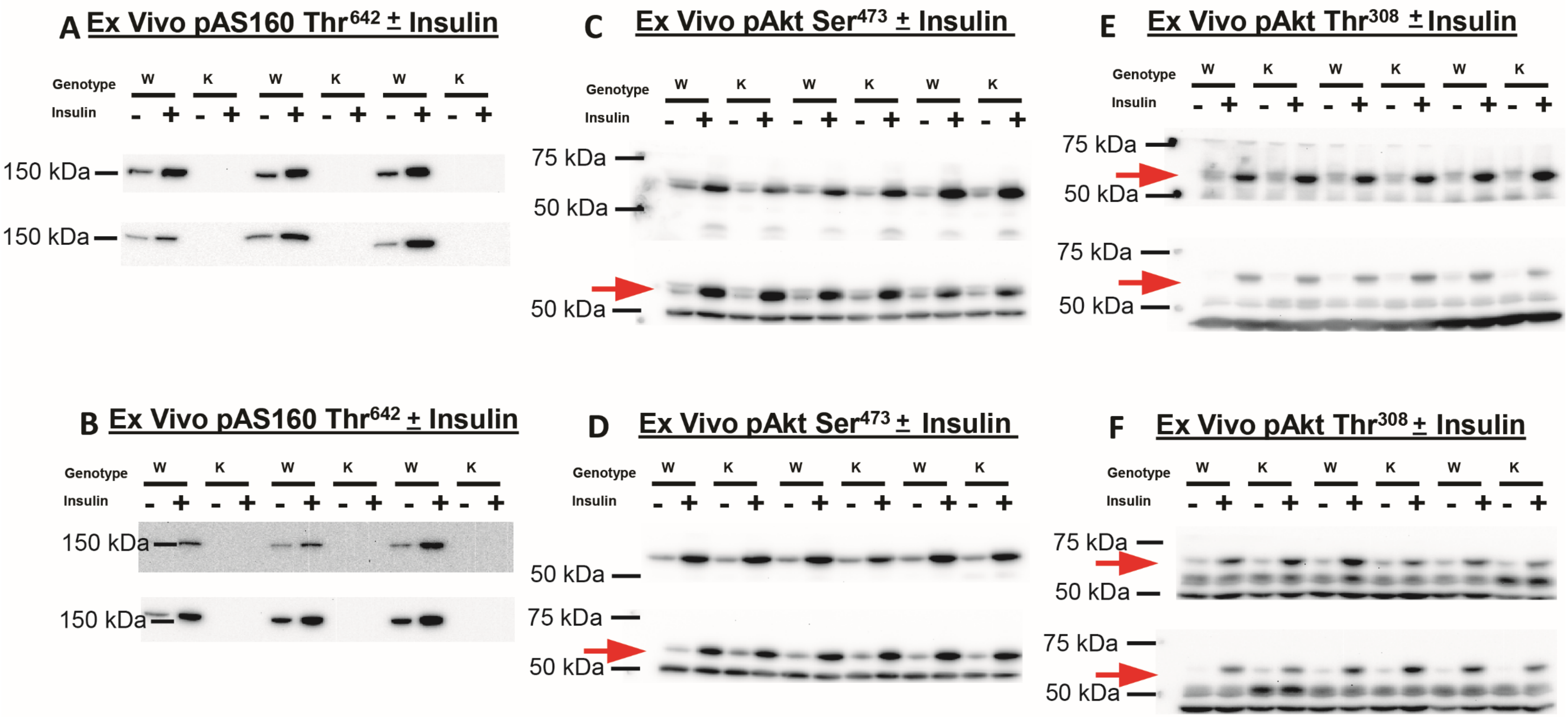

W: wild type
K: AS160 Knock-out
All images were captured using the Chemilluminescence channel by FluorChemE by Proteinsimple
Denotes quantified band at expected molecular weight

Figure 12

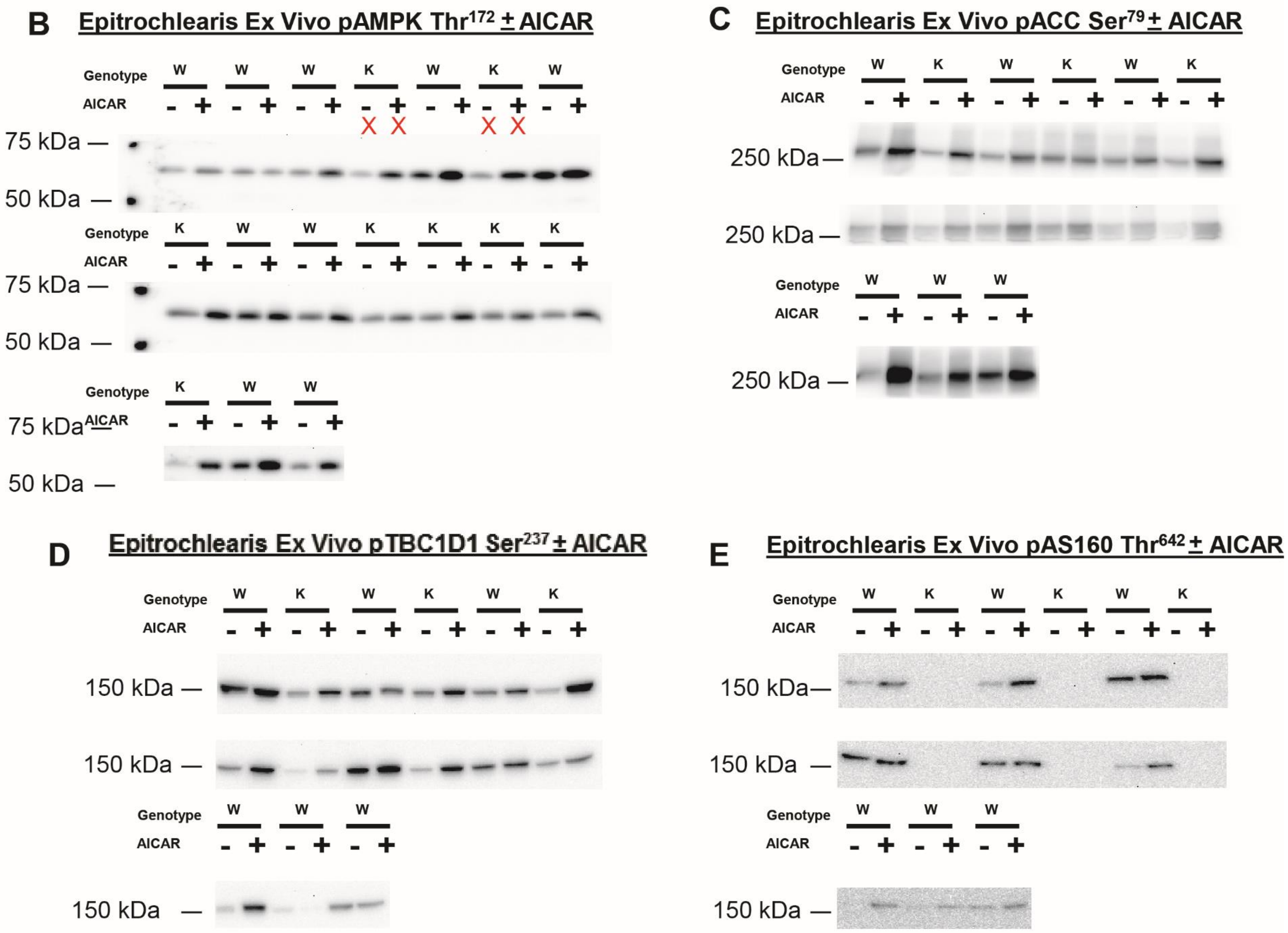

W: wild type
K: AS160 Knock-out
X: value excluded because the sample was from a male rat
All images were captured using the Chemilluminescence channel by FluorChemE by Proteinsimple

S1(All samples are from the hyperinsulinemic euglycemic clamp)

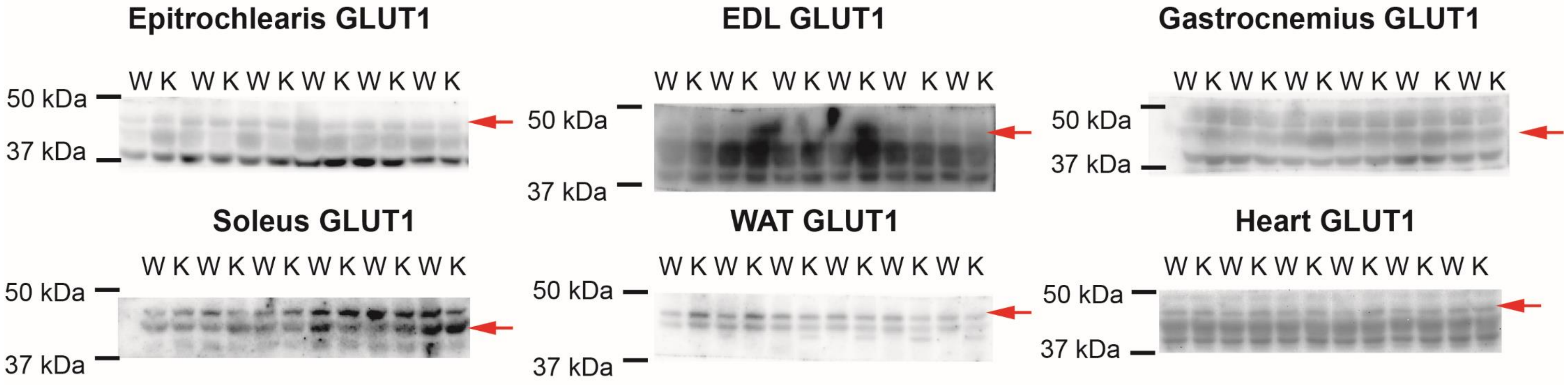

W: wild type

K: AS160 Knock-out

All images were captured using the Chemilluminescence channel by FluorChemE by Proteinsimple

← Denotes quantified band at expected molecular weight

S2 (All samples are from the hyperinsulinemic euglycemic clamp)

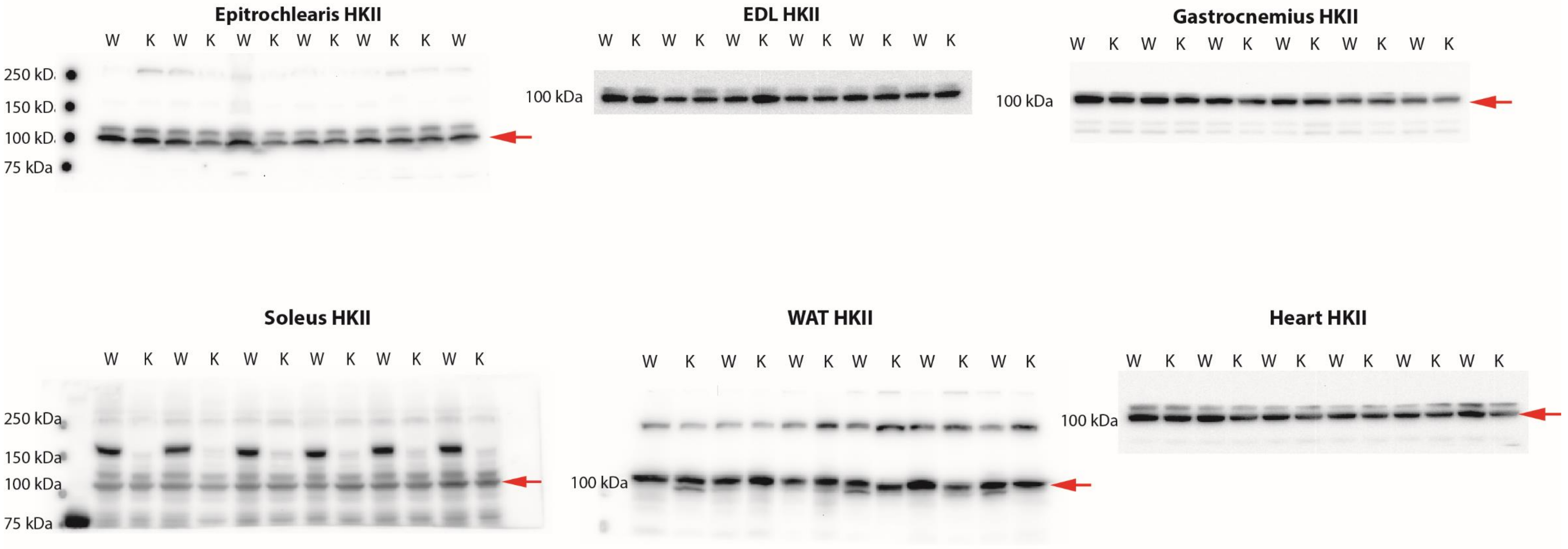

W: wild type
K: AS160 Knock-out
All images were captured using the Chemilluminescence channel by FluorChemE by Proteinsimple
← Denotes quantified band at expected molecular weight

S3 (All samples are from the hyperinsulinemic euglycemic clamp)

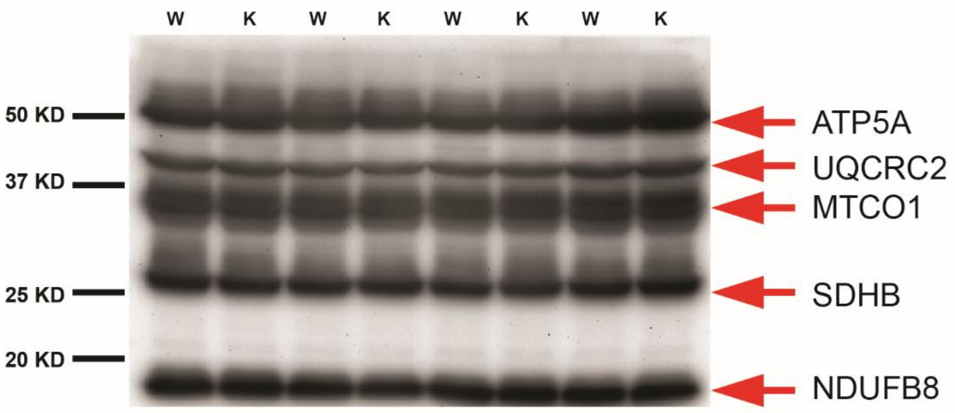

W: wild type
K: AS160 Knock-out
All images were captrued using the Chemilluminescence channel by FluorChemE by Proteinsimple
Denotes quantified band at expected molecular weight
